# Co-STARS: a feasibility evaluation of a co-produced mental health literacy training package to reduce mental health inequities for Black young people in underserved communities – study protocol for a randomised controlled trial with an external pilot, process evaluation and economic analysis

**DOI:** 10.1136/bmjopen-2025-103120

**Published:** 2026-03-18

**Authors:** Balachandran Kumarendran, Elizabeth Cherrington, Siddhartha Bandyopadhyay, Francesca Crowe, Katarzyna Karolina Machaczek, Luke Brown, Niyah Campbell, Gerald Jordan, Megan A Pope, Milan Antonović, Matthew Taylor, Nadia W, Ayan Mahamud, Joht Singh Chandan, Sian Lowri Griffiths, on behalf of the Co-STARS youth co-production group, Shanissi Okita

**Affiliations:** 1 Institute for Mental Health, University of Birmingham, Birmingham, UK; 2 Mental Health Mission Midlands Translational Centre, Institute for Mental Health, University of Birmingham, Birmingham, UK; 3 Department of Economics, University of Birmingham, Birmingham, UK; 4 Centre for Crime, Justice and Policing, University of Birmingham, Birmingham, UK; 5 Department of Applied Health Sciences, University of Birmingham, Birmingham, UK; 6 Centre for Applied Health and Social Care Research (CARe) and Advanced Wellbeing Research Centre (AWRC), Sheffield Hallam University, Sheffield, UK; 7 School of Psychological Science, University of Bristol, Bristol, UK; 8 The University of Sheffield, Sheffield, UK; 9 University of Birmingham, Birmingham, UK; 10 University of Birmingham College of Medical and Dental Sciences, Birmingham, UK

**Keywords:** MENTAL HEALTH, PUBLIC HEALTH, Pragmatic Clinical Trial, Feasibility Studies, Health Equity

## Abstract

**Introduction:**

Severe mental illness such as psychosis is among the most disabling illnesses worldwide, disproportionately affecting minoritised ethnic groups and those in socioeconomic disadvantage. In the UK, people from Black ethnic backgrounds are more likely to experience a first episode of psychosis and to be detained under the Mental Health Act than White British people. There is a clear need for mental health services to improve cultural awareness and understanding of the broader social needs of minoritised groups, as well as the need to improve mental health literacy (MHL) within Black communities to empower individuals to seek timely mental health support. This protocol describes our programme of work which aims to assess the feasibility, acceptability and cost-effectiveness of Co-STARS, which is a co-produced, culturally appropriate tiered training package.

**Methods and analysis:**

We co-produced a culturally appropriate, place-based, tiered MHL training package (Co-STARS) to deliver within underserved Black communities and via an e-learning package implemented among staff within mental health trusts. The training will be evaluated in stages. First, a pilot cluster randomised controlled trial will assess the feasibility and acceptability (defined as participants’ perceptions of the training’s relevance, usefulness and delivery) of a lived experience-led MHL training package delivered by Black young people with experience of mental ill health, to underserved communities in Birmingham, UK. Acceptability will be quantified through participation and completion rates and explored qualitatively via focus groups and interviews. Second, a stepped-wedge cluster randomised trial will evaluate the feasibility of an e-learning training programme for mental health professionals. We will embed a process evaluation to explore change mechanisms and identify barriers and enablers for future implementation. Third, we will use realist-informed participatory systems mapping and novel epidemiological analyses to explore downstream effects (ie, improved care access for Black ethno-racial groups within the intervention areas). Last, a cost-effectiveness framework will be developed to assess whether the intervention is good value for money in future efficacy trials. In the cluster trial, eight clusters will be randomised to the intervention arm (face-to-face training in the community) and control arm (display of MHL materials) with pre- and post-assessments in 120 participants from 8 clusters, 3 weeks apart. In the stepped wedge trial, six clusters (clinical teams within NHS mental health trusts) including 120 NHS staff in total, will move from control phase to intervention phase in a stepped wedge manner, with pre-assessments and post-assessments.

**Ethics and dissemination:**

This proposal was reviewed by the Research Governance of the University of Birmingham and UK Research and Innovation (UKRI) grant reviewers. Ethics approval was granted by East of Scotland Research Ethics Service. The findings will be communicated in research conferences, stakeholder meetings, via social media, through publication in peer-reviewed journals and as a policy document.

**Trial registration number:**

ISRCTN10517405.

**STRENGTHS AND LIMITATIONS OF THIS STUDY**

- The trial intervention was co-produced by Black young people with lived experience of mental ill-health, creating a culturally appropriate, place-based, tiered mental health literacy training package delivered by young people.
- Utilises a two-pronged approach aimed at improving culturally responsive care among NHS staff and enhancing mental health literacy among Black communities.
- Employs a pilot cluster randomised controlled trial and a pilot stepped-wedge trial design.
- Limitations of e-learning interventions include superficial learning and a lack of peer or interactive learning.
- Community-based training has limitations including participants’ proficiency with the English language.

## Introduction

### Background and rationale

 Severe mental illnesses (SMI) such as psychosis are among the most disabling illnesses worldwide.^1^ They are accompanied by enormous personal, healthcare and societal costs and disproportionately affect minoritised ethnic groups and those in socioeconomic disadvantage.^2 3^ Within the UK, people from Black ethnic backgrounds (African, Caribbean and British) are three to five times more likely to experience a first episode of psychosis (FEP) and are more likely to be detained under the Mental Health Act than White British people.^4^,^7^

Factors contributing to these disparities are complex and influenced by wider inequities within and beyond health systems, including bias, discrimination and racism towards Black individuals, as well as stigma and lack of recognition of the symptoms of mental illness.^8^,^10^ Further barriers to accessing timely care include internalised and externalised stigma, differing illness attributions and a disinclination to access mental health services and primary care, with a preference to seek help from faith-based or community organisations.^11^,^13^ This lack of trust and disengagement in services is thought to stem from feelings of disempowerment within the system, perpetuated by negative experiences and relationships with health professionals including experiences of racism, cultural insensitivity and care with diminished autonomy.^14^,^18^ Black individuals with psychosis are less likely to be offered National Institute for Health and Care Excellence (NICE) recommended treatments such as clozapine for treatment-resistant schizophrenia^19^,^21^ or cognitive behavioural therapy,^22^ but are more likely to be prescribed depot antipsychotics, re-admitted to hospital and to have longer inpatient stays.^19 23^ The consequences of these experiences are likely to be far-reaching, with recent work showing poorer 5-year clinical and social outcomes for those from Black backgrounds with FEP.^24^

Health literacy refers to an individual’s ability to understand and use information to make decisions about their health and navigate health services.^25 26^ It is described by the WHO as the cornerstone of a healthy society, with health literate individuals more likely to actively participate in societal economic prosperity, community activities and enjoy better health and well-being.^26^ Low levels of health literacy, often observed in individuals from lower socio-economic backgrounds, minority ethnic groups or those affected by mental health conditions, the groups already facing significant health inequities,^25 27^ are recognised by the WHO as being one of the most influential social determinants of health.^25 26 28 29^ With rising poverty in the UK, coupled with an increased prevalence of mental health difficulties, particularly in young people,^15 30^ addressing these issues has never been more pertinent.

Mental health literacy (MHL) refers to having the necessary skills, knowledge and agency to aid in the recognition, management and prevention of mental illness.^31^ MHL is essential for promoting early detection and early treatment of mental health problems to improve longer-term outcomes.^32^,^34^ In large-scale programmes that seek to reduce mental health stigma, interventions developed and delivered by people with lived experience of mental health conditions were the most effective.^35 36^ Thus, there are calls for MHL interventions to be developed and applied so that they fit the context in which they are to be deployed, and that high priority groups are engaged through co-design principles.^26 37^ To date, there is no evidence of effective MHL interventions delivered by (and for) high priority, marginalised groups, with research in this area limited to low-quality studies and lack of rigour to establish efficacy.^3538^,^41^

### Co-STARS: a co-produced training package

Building on the findings from Phase 1 of the Co-STARS programme (2023–2024), which involved the co-production of the training package with Black African and Black Caribbean young people with lived experience of mental ill health, we will conduct a feasibility evaluation of a co-produced, culturally appropriate, place-based MHL training package (Co-STARS) delivered within underserved communities across Birmingham, UK, and via an e-learning package implemented within two large mental health trusts serving these communities. Birmingham is a large multi-cultural city with numerous risk factors indicative of low levels of health literacy. 51% of the population consists of individuals from ethnic backgrounds, with 9% from Black African and Black Caribbean heritage (compared with 3.5% as national average).^42^ Approximately half of Birmingham wards fall within the most deprived decile of the English Index of Multiple Deprivation, indicating that these areas are among the top 10% of neighbourhoods nationally with the highest levels of social and economic disadvantage.^42^ The combined effects of deprivation and minority status are reflected in the high estimated incidence of psychosis in Birmingham, with approximately 431.2 new cases per 100 000 young people aged 16–35 years annually, of which around 75% occur among individuals from ethnic minority backgrounds, according to modelled forecasts from PsyMaptic-A.^43 44^ Birmingham is also one of the most vulnerable areas to literacy challenges in the entire country.^45^ These highlight the importance of addressing MHL in settings such as Birmingham to address place-based health inequities.

By strengthening both community MHL and professionals’ cultural responsiveness, Co-STARS seeks to improve recognition of emerging symptoms, reduce stigma and mistrust and promote timely help-seeking. Over time, these mechanisms are expected to contribute to earlier access to care, reduction in crisis presentations and better recovery trajectories among Black young people at risk of psychosis. Indicators of success in this feasibility phase include improvements in mental health knowledge, stigma reduction and help-seeking efficacy (WP1) and enhanced multicultural competence among professionals (WP2).

### Project phases

**Figure 1:**
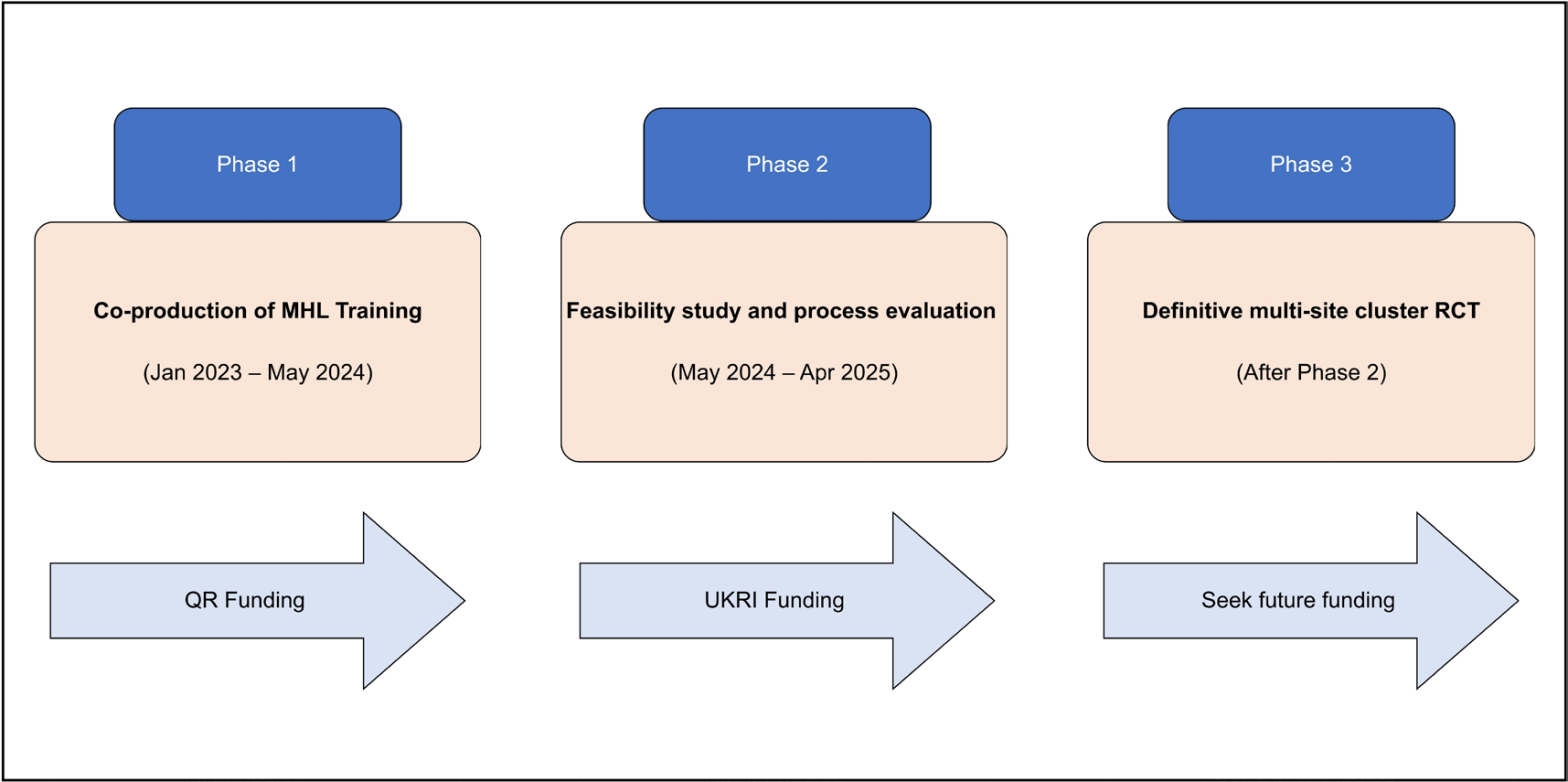
Phased approach to Co-STARS evaluation. This figure outlines the three planned phases: Phase 1 (co-production of MHL training), Phase 2 (feasibility evaluation) and Phase 3 (definitive multi-site trial). MHL, mental health literacy; RCT, randomised controlled trial; UKRI, UK Research and Innovation.

This is the second phase of a recently completed project (phase 1; figure 1), developed in line with the Medical Research Council framework for developing and evaluating complex interventions.^46^ Phase 1 of Co-STARS involved the co-production of the training package with young people from Black African and Black Caribbean backgrounds with lived experience of mental ill health. Interviews and focus groups with mental health professionals were also conducted to assess existing knowledge and identify areas where training interventions were necessary. Phase 1 of the Co-STARS programme (2023–2024) focused on the co-production of the training content and format through workshops and focus groups with Black young people, mental health professionals and community leaders. This formative phase identified key barriers to equitable care and generated the two training components evaluated in the current Phase 2 feasibility study (WP1 and WP2).

The following outputs from the first phase will now be evaluated in phase 2:

- Lived experience-led training package delivered by young people within Black diaspora communities (evaluated as part of WP1).
- e-learning on culturally responsive care for mental health professionals (evaluated as part of WP2).

**Figure 2:**
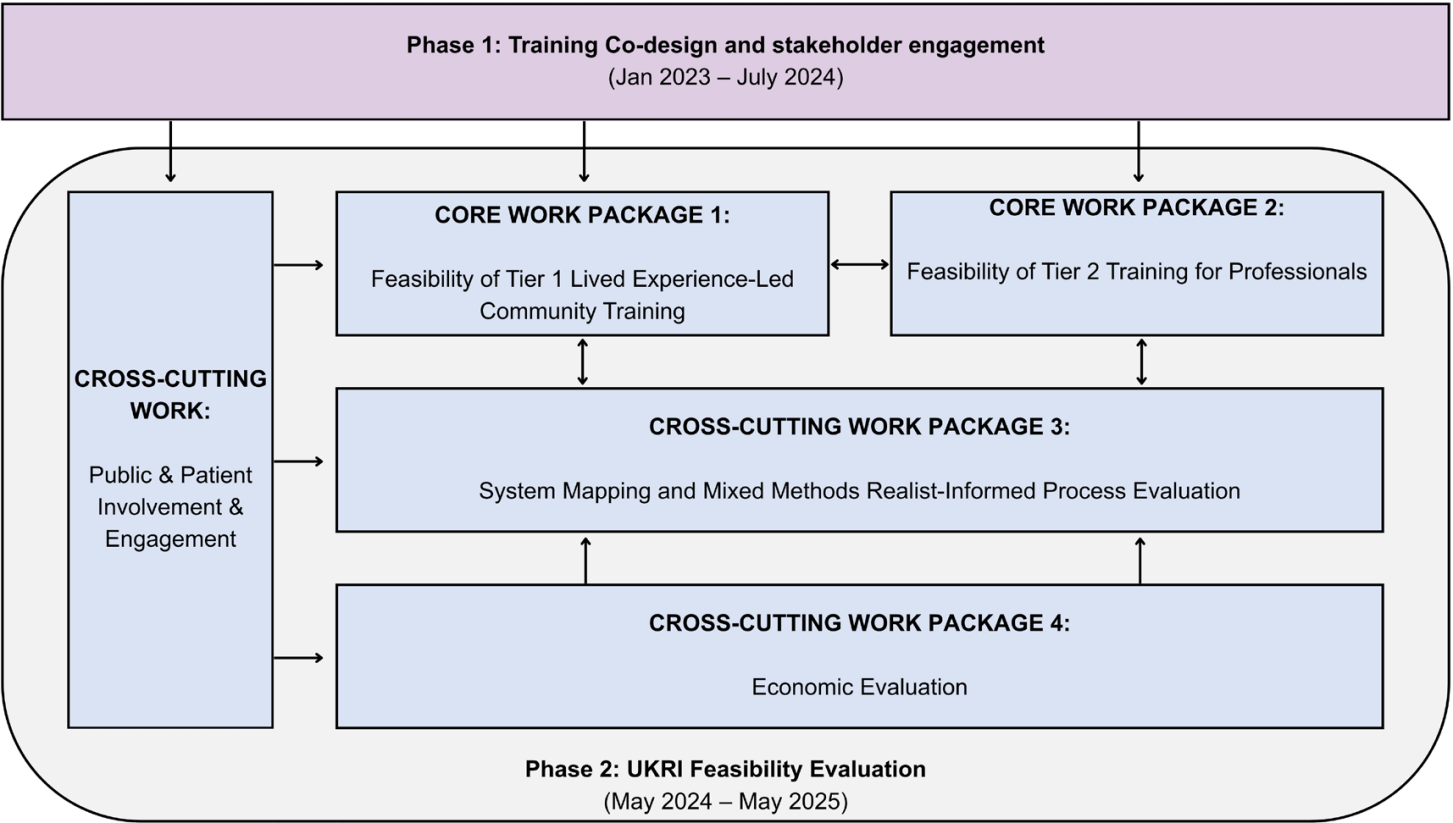
Overview of work packages for Phase 2 feasibility evaluation development. This diagram summarises the four work packages and cross-cutting components, including public involvement, systems mapping and economic evaluation. UKRI, UK Research and Innovation.

We will pilot the feasibility of the training outputs through a series of evaluations (figure 2), which will form the basis for our Phase 2 activities (figure 1). If we demonstrate acceptability and feasibility of the training package, we will seek funding to progress to Phase 3, which is a multi-site (across all four nations in the UK) large scale cluster randomised controlled trial (CRT).

## Methods and analysis

Trial status: Phase 2 trial recruitment started in March 2025 and is due to conclude in January 2026.

Phase 2 aims: We aim to undertake a pilot evaluation assessing the feasibility, acceptability and cost-effectiveness of a MHL package designed to promote equitable mental healthcare access and improve outcomes for underserved groups. We co-designed and co-developed the following work packages (WP; figure 2) based on close engagement with stakeholders and patient and public involvement and engagement (PPIE) :

- WP1: Pilot CRT of lived experience-led MHL training in community settings.
- WP2: Pilot Stepped Wedge Cluster Randomised Trial (SWCRT) of e-learning for mental health professionals.
- WP3: Mixed-methods study: Systems mapping and systems change evaluation.
- WP4: Economic evaluation of WP1-3 to develop a framework to examine cost-effectiveness and social return on investment (SROI).

In addition to assessing impact, we will embed a mixed-methods realist-informed process evaluation to explore the mechanisms of change and resources required to support intervention reach and impact in the continued rollout. We will develop theories that explain how outcomes targeted by Co-STARS are achieved, for whom, why and under what circumstances (contexts). The process evaluation will comprise targeted searches for literature, using the principles of a realist synthesis, interviews with various groups of stakeholders and a document review. The process evaluation will be critical to understand how the implementation is achieved, how each of the interventions produces change and how context might affect implementation and outcomes, including impact on mental health inequalities. Although effectiveness will not be tested at this stage, a pilot randomised design is essential to evaluate feasibility of recruitment, randomisation, data collection and implementation procedures of this intervention. These feasibility outcomes will determine whether progression to a definitive multi-site effectiveness trial is justified and inform its design.

A completed SPIRIT 2025 checklist is provided as online supplemental material 1, detailing the corresponding page and line references within this protocol.

### Co-production and cultural considerations

Young people from Black African and Black Caribbean-diaspora backgrounds were actively involved throughout the co-production of this study. Discussions with participants highlighted that ‘Black’ is not a single ethnicity or culture, and experiences vary widely depending on the intersections of social identities. These reflections informed the development of culturally responsive training, emphasising the diversity within Black communities and the importance of recognising intersecting social identities. The co-production process ensured that the study design, measures and materials were sensitive to participants’ cultural and social contexts. The co-production extended beyond the study design to include co-development of the training content, delivery format and visual and verbal materials used within both the community and e-learning packages. Young co-researchers also contributed to piloting and refining outcome measures and feedback procedures.

#### WP1: pilot CRT of lived experience-led MHL training in community settings

Research question: Can a lived experience-led, community-delivered MHL training package be feasibly implemented and accepted within underserved Black communities?

Design: Pilot CRT assessing feasibility, acceptability and preliminary change in mental health knowledge, attitudes and help-seeking.

Objective: To assess the feasibility and acceptability of the MHL intervention delivered to underserved communities and assess the barriers and enablers to support the future implementation strategy/trial.

Design: An unblinded, pragmatic, feasibility CRT implemented within a specific underserved community in Birmingham. We will adhere to the CRT consort extension^47^ to ensure transparency and quality of reporting. A mixed methods realist approach will be adopted for the process evaluation.

Method: Recruitment of clusters and implementation of the intervention will occur over a 6-month time frame (months 4–9). Participants within the cluster will be consented and asked to complete pre-outcome/post-outcome measures. We will also conduct structured observations assessing the fidelity of intervention delivery, as well as two focus groups and semi-structured interviews with participants within the control and intervention arms, as well as young people delivering the training.

Data collection schedule: Baseline data will be collected immediately prior to training (T0), and follow-up data 3 weeks post-intervention (T1). Research assistants trained in Good Clinical Practice will oversee all data collection, supervised by the principal investigator.

Setting: We will target two constituencies in the West region of Birmingham and Black Country region. The West of Birmingham is a region with the highest proportion of Black African and Black Caribbean individuals in Birmingham (19% compared with 9% and 3.5% in Birmingham and UK, respectively), with over half of the population living in the top 10% decile for deprivation, meaning that these communities are likely exposed to a multitude of social risk factors for development of severe mental illness.^42^

Unit of randomisation: Middle layer super output areas (MSOAs) within Birmingham and the Black Country will serve as the units of randomisation (clusters) and will be randomly allocated on a 1:1 basis to receive the intervention or control. Each MSOA represents a geographically defined area with a population of approximately 5000–15 000 residents, providing an appropriate ecological unit for community engagement and potential linkage with routinely collected data in future phases.

Within each selected MSOA, the intervention will be implemented in community settings such as places of worship (eg, Black-majority churches), youth centres, community centres and youth residential settings that already serve local Black communities. To ensure feasibility and representativeness, we restricted the sampling frame for cluster randomisation to MSOAs with intermediate Black population density, based on the proportion of residents identifying as Black (African, Caribbean or Black British) in the 2021 Census. This restriction avoids areas where community engagement would be unfeasible (very low-density MSOAs) and reserves very high-density MSOAs for the future definitive trial. Importantly, if recruitment and delivery are feasible in intermediate-density MSOAs, it is likely to remain feasible in MSOAs with higher Black population density, providing a conservative test of feasibility.

Randomisation will use restricted (covariate-constrained) allocation to maintain approximate balance on (i) area-level deprivation and (ii) anticipated participant numbers (based on venue capacity and local population). The randomisation sequence will be generated by an independent researcher/statistician prior to implementation.

By design, concealing treatment allocation is not possible in this community-based feasibility study. Given the nature of the training intervention, blinding of participants and researchers is not feasible. However, the primary feasibility outcomes (recruitment and retention rates, measure completion and acceptability) are objective and unlikely to be influenced by allocation awareness.

Population: Participants aged 18–65 years, self-identifying as members or regular attendees of community settings within participating MSOAs, will be eligible. Inclusion criteria include age 18–65 years, residence within the selected MSOA and ability to provide informed consent. Exclusion criteria include inability to consent due to cognitive impairment or severe current psychiatric crisis. Researchers will recruit participants through community champions and youth networks, provide study information sheets and obtain written informed consent prior to baseline assessment.

Intervention: The intervention is the lived experience-led MHL training. The training will be delivered by young people of Black African and Black Caribbean diaspora, lasting approximately 1.5 hours and will cover: (1) symptoms and signs of mental illness (including SMI); (2) how to distinguish mental health symptoms from normal behaviours; (3) attitudes and beliefs about people with mental illness; (4) advice on how to manage well-being; and finally, (5) information about local services and help-seeking. The intervention will be reported using Template for Intervention Description and Replication (TIDER).^48^

Control: Written MHL material (information leaflets and posters) placed within the community setting. Prior to these materials being placed in the community setting, participants will be invited to complete baseline measures and then re-approached after 3 weeks to collect follow-up measures.

Outcome: Feasibility outcomes include proportion of consenting clusters, percentage of training uptake and completion of pre-outcome and post-outcome measures, as well as the acceptability of the intervention and assessment of the barriers and enablers. Our secondary outcome is the assessment of key training outcomes: (1) *Knowledge*—The Mental Health Knowledge Scale^49 50^; (2) *Illness attributions and stigma*—Reported and Intended Behaviour Scale: Intended Behaviour Subscale to assess intended and reported stigmatising behaviours and desire for social distance from someone with a mental illness^51^; Community Attitudes towards Mental Illness scale^52^; and (3) *Help-seeking attitudes and efficacy*—General Help-Seeking Questionnaire.^53 54^ All instruments used have demonstrated robust psychometric properties in previous MHL research, including good internal consistency (Cronbach’s α>0.70) and construct validity.^49^,^54^

Sample size: We will recruit 120 participants from the eight randomised clusters. Formal power calculations are not necessary as no hypothesis testing for trial effectiveness is being evaluated.^55^ A sample of 120 (with ~60 per arm) meets sample size recommendations for pilot and feasibility studies and is sufficient to provide precision of feasibility parameters and estimates of study summary measures for a definitive trial.^56^ 20 participants in total will be recruited for focus groups (three groups of five diverse participants) and five 1:1 interviews.

Analysis: Descriptive statistics will be used to assess training fidelity and feasibility outcomes. Other data will be analysed using thematic analysis to generate important themes and patterns.^57 58^ For the secondary outcomes, means, CIs, intra-cluster correlation coefficient (ICC) of the outcome measures (both within-study and between-study clusters) will be estimated to determine which outcome(s) are most sensitive to change and provide sample size calculation for definitive trial.

ICCs for each quantitative outcome will be estimated using mixed-effects regression models including a random intercept for clusters, following the approach recommended by Hemming *et al* for cluster and stepped-wedge feasibility studies.^59^ Analyses will be undertaken in Stata (V.18). The ICC estimates, along with observed variances and completion rates, will inform the design of the definitive trial by (i) identifying outcomes that are most sensitive to change and (ii) providing parameter estimates for sample size and power calculations.

Missing data will be assessed for extent and pattern. The proportion of missing values for each variable will be reported, and exploratory analyses will assess whether missingness appears random or systematic. As this is a feasibility study, no formal imputation will be undertaken for primary feasibility outcomes; however, simple sensitivity analyses using mean or regression-based imputation may be conducted to evaluate the potential impact of missing data on secondary outcomes.^55 56^ The findings will inform strategies for minimising and handling missing data in the future definitive trial.

#### WP2: a pilot SWCRT of e-learning for mental health professionals

Research question: Is an e-learning module on culturally responsive care feasible and acceptable for delivery to NHS mental health professionals?

Design: Pilot stepped-wedge cluster randomised trial assessing feasibility, uptake and change in multicultural competence scores.

Aims and objectives: To evaluate the feasibility and acceptability of the e-learning to inform our implementation evaluation in a future trial (phase 3).

Design: We will adopt a pragmatic SWCRT^60^ design with random and sequential crossover of clusters (youth mental health teams) from control to intervention (our e-learning module) until all clusters are exposed. Because training is delivered openly within existing clinical teams, outcome assessment cannot be blinded; feasibility endpoints are objective and carry minimal risk of bias.

**Figure 3:**
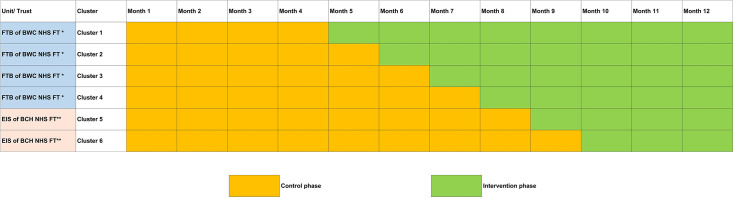
Co-STARS e-learning stepped-wedge rollout. This schematic shows the sequential rollout of the e-learning intervention across six clusters over the 12-month study period. *Forward Thinking Birmingham of Birmingham Women’s and Children’s NHS Foundation Trust (FTB of BWC NHS FT). **Early Intervention Services of Black Country Healthcare Foundation Trust (EIS of BCH NHS FT).

Methods: There will be a phased and sequential implementation of the e-learning at regular intervals across teams and trusts over a 6-month period (months 4–9; figure 3). Staff will complete the training outcome measure before and after the e-learning.

The training will be deployed in a staggered manner in line with the stepped wedge approach. Starting in the first Mental Health Trust (Forward Thinking Birmingham (FTB) of Birmingham Women’s and Children’s NHS Foundation Trust; please see setting for description) in month 4 and rolled out to the four teams in staggered intervals. The training will then be rolled out in the second Mental Health trust (Early Intervention Services (EIS) of Black Country Healthcare NHS Foundation Trust) with staggered implementation between months 6–9. Through the course of implementation, we will conduct staff focus groups and semi-structured interviews. Baseline (pre-training) and post-training (4 weeks later) data will be collected electronically via the NHS Learning Hub platform.

Setting: FTB, part of the Birmingham Women’s and Children’s NHS Foundation Trust, is a 0–25 years community and inpatient mental health service and is the primary mental health service regionally for this population. Within FTB, there are four specialised care pathways, including Youth Justice, Specialist Eating Disorders and Core mental health team and EIS, which offer intensive community support to young people with a FEP.

The second trust is Black Country Healthcare NHS Foundation Trust, in which we will target four EIS teams (Dudley, Walsall, Sandwell and Wolverhampton), which provides community support to young people with psychosis between the ages of 14 and 65 years.

Randomisation: Cross-over of clusters (clinical team; n=6) from control to intervention will occur approximately every 4 weeks.

Population: *Inclusion criteria*: NHS staff working within participating teams. *Exclusion criteria:* administrative personnel not directly engaged in mental healthcare delivery. Recruitment will occur via internal staff communications and service managers. A range of professionals (n=120) will be expected to complete the training, including psychiatrists, psychologists, community psychiatric nurses, occupational therapists, support workers and social workers. A purposive sampling method will be used to recruit staff to the focus groups and 1:1 interview, ensuring a balance of staff backgrounds, experience and representation from across different teams and trusts. We will also adhere to INCLUDE guidelines to ensure diversity and inclusion in our sampling.^61^

Intervention: The intervention is a 60 min e-learning module targeted at professional systems and public organisations involved in mental healthcare pathways. This e-learning is hosted by the NHS Learning Hub platform. Informed by lived experience, the e-learning seeks to raise awareness of sociocultural diversities, awareness of cultural barriers, multicultural knowledge and sensitivity and responsiveness to patients from Black ethno-racial backgrounds.

Control: The control condition will be the unexposed observation period before sequentially crossing over to the exposed observation period (receiving the intervention).

Outcome: Feasibility process outcomes will include training uptake, defined as the proportion of eligible staff completing the e-learning module within each cluster, and acceptability, assessed quantitatively through completion and satisfaction rates and qualitatively via staff focus groups and interviews exploring the training’s perceived relevance, usefulness and delivery.^62^ The qualitative component will be guided by the Acceptability of Intervention Measure framework. Secondary outcome will be assessed using the California Brief Multicultural Competence Scale (CBMCS),^63 64^ which assesses mental health staff knowledge, attitudes, skills and competencies when working with minoritised groups. The CBMCS has shown strong reliability (Cronbach’s α=0.91) and construct validity across diverse healthcare professional samples.^63 64^ Pre-training and post-training CBMCS scores will be compared at individual and cluster levels to estimate mean change and ICCs.

Sample size: Formal power calculations are not necessary as no hypothesis testing for trial effectiveness is being evaluated.^55^ A sample of 120 meets sample size recommendations for pilot and feasibility studies and is sufficient to provide precision of feasibility parameters and estimates of study summary measures for a definitive trial.^56^

Analysis: Descriptive statistics will summarise training uptake, completion rates and acceptability indicators for each cluster and step period. Quantitative comparisons of pre-training and post-training CBMCS scores will be undertaken using mixed-effects linear regression models with a random intercept for cluster and a fixed effect for time step, following Hemming *et al*’s recommendations for stepped-wedge feasibility trials. Analyses will be performed using Stata (V.18).

Qualitative acceptability data will be analysed thematically to identify perceived enablers and barriers to implementation.

Estimates of outcome variability, ICCs and temporal trends will be reported with 95% CIs to inform sample size and power calculations for the definitive trial.

#### WP3: mixed-methods study: systems mapping and systems change

Research question: What system-level mechanisms link the Co-STARS intervention to improved access and outcomes for Black young people?

Design: Realist-informed mixed-methods study combining participatory systems mapping, Delphi consensus and exploratory time-series analysis.

We will conduct a mixed-method, realist-informed process evaluation to support a proposed whole-systems approach for assessing the impacts of the training model described in WP 1 and 2. This process evaluation will explore *how*, *why*, *for whom* and *under what circumstances* the training may generate whole systems change (online supplemental material 1). We will further explore system-level mechanisms.

Systems map: A systems map^65^ will be developed in three phases: (1) First, we will undertake a concept mapping workshop involving key stakeholders (patients, research team, providers and policymakers). This will be guided by a draft model produced by the team (who consist of mental health experts in the region) which will then be introduced and amended using an iterative consensus building process in a face-to-face workshop. (2) This will be followed by a modified two-stage modified Delphi survey inviting regional mental health experts to provide their agreement or disagreement with the systems map and willingness to be involved in semi-structured interviews with the research team. (3) Those willing to take part in interviews will then be invited to share their opinions on how the intervention may impact the system map and in particular their opinion on potential outcomes which should be measured at a system-wide level.

Exploring the early impacts of co-STARS: To identify whether the intervention is providing tangible impacts on a system-wide level, we are proposing to pilot a data-driven impact evaluation. We are planning to undertake an interrupted time series (ITS) to ascertain whether the introduction of the intervention leads to a change in the relevant outcomes identified in the systems mapping process. This type of analytical approach has been advocated for use in public health evaluations and is related to structural break modelling.^66^

This pilot evaluation will be undertaken in three phases: (1) Identification of suitable data sources: Guided by health data science expertise in the research team, expert opinion from the systems mapping process and through open source searching, we aim to compile a directory of suitable datasets and outcomes which could inform the systems wide impact of the intervention. For example, West Midlands Police Data will be used to examine the rate of detentions under Section 136 of the UK Mental Health Act among Black males. These detentions represent police-initiated transfers of individuals appearing mentally unwell from public settings to a place of safety for clinical assessment. Data sources will include open-access administrative datasets (eg, NHS Digital, West Midlands Police s136 statistics) and local health service indicators, each evaluated for completeness and temporal resolution before inclusion. (2) Trial of ITS model: Using available datasets (those open access or those already accessible by the research team), we will undertake a pilot ITS. The study period will be set between 2022 and 2024. Incidence rates (calculated by the number of new cases divided by the at-risk population) will be analysed on a monthly basis (weekly where possible) throughout the study period and these trends will be depicted graphically. The primary break point that will be examined is the introduction of the intervention; thus, monthly data 1 year prior and 1 year following this timepoint will be included. We will examine the sensitivity of the analysis around this break point. (3) Stakeholder engagement: We plan to undertake three focus groups (consisting of six to eight people) with key stakeholders (patients, providers and commissioners) to demonstrate our findings from the trial to unpick the possible mechanisms for these changes as a result of the impact of the intervention of the systems map. The secondary aim of these focus groups will be to optimise the strategy to undertake such systems change evaluation of a broader scale when the intervention is rolled out for a full trial.

#### WP4: economic evaluation to examine cost-effectiveness and SROI

Research question: What are the costs and potential cost-consequences of implementing the Co-STARS tiered training approach?

Design: Economic evaluation and SROI analysis based on feasibility trial data.

Our approach is likely to reduce direct health costs (admission and healthcare usage), but it could also have important cost implications for the healthcare sector, public sector and voluntary sectors. The primary outcome for the economic evaluation will be the incremental cost per unit improvement in MHL (community participants, WP1) and multicultural competence (NHS staff, WP2). Secondary outcomes will include changes in rate of mental health incident rates, quality of life, healthcare utilisation and social value generated, assessed using a SROI framework. The primary base case analysis will adopt a public sector perspective in line with NICE guidelines, with a wider societal perspective explored as a secondary analysis. Uncertainty in cost and outcome estimates will be addressed using deterministic and probabilistic sensitivity analyses, with results presented as 95% CIs and cost-effectiveness acceptability curves. This feasibility-stage economic evaluation will not test intervention effectiveness; rather, it will develop a cost-effectiveness framework and examine the practicality of collecting cost and outcome data within pilot conditions. Preliminary cost-consequence estimates using the data collected in line with the framework developed will be used to inform the design and analytic framework of the definitive Phase 3 trial.

Data collection: First, and most relevant to WP1 and WP2, resource use data will be used to estimate the costs associated with each of the intervention and control arms. This will include (i) intervention costs; (ii) healthcare resource use; (iii) wider public sector resource use; and (iv) private costs. Information on unit costs or prices will be sourced to attach to each resource use item, to enable an overall cost to be calculated (eg, Personal Social Services Research Unit (PSSRU) Unit Costs of Health and Social Care).

Cost-effectiveness: To compare intervention arms with the control, a within-study analysis and a model-based economic analysis will be undertaken. This will primarily use the data collected within the trial. Initially, the base case analysis will be framed in terms of a cost-consequences analysis, and data will be reported in a disaggregated manner on the incremental cost and important consequences assessed in WP1 and WP2. The main economic analysis will assess the feasibility of undertaking a cost-effectiveness analysis based on the costs and health outcomes collected in WP1 and WP2. The economic evaluation will be conducted and reported in accordance with relevant guidelines (eg, Consolidated Health Economic Evaluation Reporting Standards (CHEERS) checklist) and recommended methodologies.^67 68^ If this is found to be feasible, this will drive the cost-effectiveness analysis for a trial and future real-world settings if the intervention is widely adopted.

SROI: As not all costs are quantifiable, we will also explore an SROI.^69^ Our youth advisory group (and work undertaken during phase 1) will help us determine which items (including ones where costs are intangible) matter and discuss how they should be quantified. This is particularly relevant when considering the wider system impacts in response to the intervention (eg, the impacts of improved community connectedness).

### Patient and public involvement and engagement

This programme of work from conception through to delivery has been informed by PPIE, including young people with lived experience and our charitable partners who have provided advice on study management, training and support for PPIE, as well as inputting into the lay summary. This study represents phase 2 of a three-phase project, and engagement with young people with lived experience of mental health difficulties began during earlier consultation work and the QR-funded phase (phase 1). Young people from Black African and Black Caribbean diaspora communities contributed to shaping the research questions, intervention design and evaluation approach. Co-production workshops informed the selection of outcome measures, including acceptability of training, cultural responsiveness and community engagement outcomes. Public contributors also helped develop recruitment materials (eg, flyers and wording), training content and study procedures, and advised on the feasibility and participant burden of study procedures, including questionnaire length, training duration and follow-up interviews.

We have ensured good practice in line with UK Standards for Involvement.^61 70^ We have also appointed a diverse youth advisory group who will work closely with the research team to inform on project ethics, delivery and dissemination. The group consists of young people from Black African and Black Caribbean diaspora residing in Birmingham, and who have lived experience of mental ill health. We have also established a Research Advisory Committee comprising external researchers and relevant community stakeholders, including Community Network Support Officer, NHS Trust staff, faith leaders, representatives of Birmingham Voluntary Services Council and West Midlands Violence Reduction Partnership.

In phase 2, persons with lived experience will also contribute to the delivery of the MHL training alongside the research team. Persons with lived experience are included as co-authors and contributors to this protocol and will continue to contribute to interpretation and dissemination of findings through youth advisory meetings, community workshops and partner organisations, including voluntary sector and NHS networks. Youth contributors will be reimbursed for their involvement.

## Ethics and dissemination

This study is implemented as four work packages. The participants in all work packages will receive a participant information sheet and informed consent form prior to giving their informed written consent. The participants have the right to refuse to participate or to withdraw their consent at any time after giving their consent. The participant information sheet includes an introduction to this research, reason for the selection of participants, participants’ involvement in research, potential benefits and risks of taking part in this research, reimbursement of time and expenses, information on the organisers, insurers, funders, reviewers of this study, confidentiality, use of personal information and sources to get additional information. This also includes a detailed account on whom to report any problems. All quantitative data will be entered into secure University of Birmingham servers with restricted access. Identifiable information will be stored separately from research data, and qualitative recordings will be pseudonymised and transcribed using approved university services. Data monitoring and coding will follow the Data Protection Act 2018 and General Data Protection Regulation (GDPR) compliance.

### Research ethics approval

Ethics approval was granted by East of Scotland Research Ethics Service (EoSRES) with REC reference: 24/ES/0030, and Protocol number: RG_23-166.

### Protocol amendments

Any amendments to the protocol will be submitted to the Research Governance of University of Birmingham (ie, sponsors) for review, submitted as an amendment to study Research Ethics Committee (REC) and updated in the clinical trial registry.

### Dissemination

The findings will be communicated in lay and academic conferences, stakeholder meetings and via social media and published in peer-reviewed journals and as policy documents.

## Supplementary material

10.1136/bmjopen-2025-103120 online supplemental file 1
