# Supplementary material for: Co-STARS: a feasibility evaluation of a co-produced mental health literacy training package to reduce mental health inequities for Black young people in underserved communities – study protocol for a randomised controlled trial with an external pilot, process evaluation and economic analysis
Source: BMJ Open. 2026 Mar 18;16(3):e103120. doi: 10.1136/bmjopen-2025-103120 (PMC13007151; doi:10.1136/bmjopen-2025-103120)
Supplement: online supplemental file 1 [file bmjopen-16-3-s001.docx]

Co-Stars: A Feasibility Evaluation of a Co-Produced Mental Health Literacy Training Package to Reduce Mental Health Inequities for Black Young people in Underserved Communities: study protocol for a randomised controlled trial with an external pilot, process evaluation and economic analysis

**SPIRIT 2025 Checklist**

| **Item** | **Description of How Addressed in Co-STARS Protocol** | **Page / Line Reference** |
| --- | --- | --- |
| 1a. Title | Full title specifies design, population, and intervention: Co-STARS: A Feasibility Evaluation of a Co-Produced Mental Health Literacy Training Package to Reduce Mental Health Inequities for Black Young People in Underserved Communities. | p. 1, lines 1–4 |
| 1b. Structured summary | Cluster randomised feasibility design (WP1, 6 clusters) and stepped-wedge pilot (WP2, 6 clusters – 4 FTB, 2 BCHFT). | p. 1–2, lines 5–40 |
| 2. Protocol version | Version 2 dated 16 Oct 2025 shown in header and file name. | Title page |
| 3a. Roles and responsibilities | Names, affiliations, author contributions listed in Author Affiliations and Contributions. | p. 17–18 |
| 3b–3c. Sponsor / funder roles | Sponsor – University of Birmingham; Funders – UKRI and QR Fund; roles in Funding section. | p. 17–18 |
| 3d. Committees / coordination | Oversight by UoB Research Governance and Research Advisory Committee (includes community representation). | p. 16–17 |
| 4. Trial registration | ISRCTN 10517405 listed under Abstract and Ethics. | p. 2 and  p. 16–17 |
| 5. Protocol and SAP access | Full protocol to be made public on BMJ Open publication; SAP summarised under each WP and aligned with SPIRIT 2025. | Methods and Analysis  (p. 7–16) |
| 6. Data sharing | Data management plan approved; de-identified data and code available on request after publication. | p. 16–17 |
| 7a–7b. Funding & conflicts | Funded by UKRI and QR; conflict declared for GJ only. | p. 17–18 |
| 8. Dissemination policy | Findings shared through conferences, stakeholder events, social media, open access publications. | p. 16–17 |
| 9a. Background / rationale | Background on psychosis inequities and rationale for MHL intervention in Introduction. | p. 2–7 |

| **Item** | **Description of How Addressed in Co-STARS Protocol** | **Page / Line Reference** |
| --- | --- | --- |
| 9b. Explanation for comparator | Comparator = written MHL materials (WP1) and control observation phase (WP2). | p. 9–12 |
| 10. Objectives | Overall and WP-specific objectives summarised under ‘Aims and Objectives’. | p. 7–9 |
| 11. Patient / public involvement | Young people with lived experience involved in co-production, design, and dissemination. | p. 15–16 |
| 12. Trial design | Phase 2 feasibility evaluation: pilot cluster RCT (WP1), stepped-wedge cluster RCT (WP2), systems mapping (WP3), economic evaluation (WP4). | p. 7–9 |
| 13. Setting | Community and NHS settings in Birmingham and Black Country (FTB and BCHFT). | p. 9–12 |
| 14a. Eligibility – participants | Adults 18–65 (WP1); NHS staff (WP2); criteria in each WP. | p. 9–12 |
| 14b. Eligibility – sites/staff | Community and clinical clusters serving Black communities; trained facilitators deliver training. | p. 9–12 |
| 15a. Intervention / comparator | WP1 – 1.5 h community training vs MHL leaflets; WP2 – 60 min e-learning vs control phase. | p. 9–12 |
| 15b. Modification criteria | Participants may withdraw any time; no dose modifications required. | p. 16–17 |
| 15c. Adherence strategies | Attendance logs and completion checks for adherence. | p. 9–12 |
| 15d. Concomitant care | No restrictions on usual care or education. | p. 16–17 |
| 16. Outcomes | Feasibility and acceptability primary; secondary = MAKS, RIBS, CAMI, GHSQ, CBMCS; AIM (Weiner 2017); CHEERS 2022. | p. 10–13 and  p. 15–16 |
| 17. Harms | Low-risk educational trial; adverse events handled per ethics procedures. | p. 16–17 |
| 18. Participant timeline | Recruitment May 2024–Apr 2025; timeline and phases shown in Figures 1–2 and GANTT chart. | p. 7–9 and 12 |
| 19. Sample size | 120 participants (WP1 + WP2); rationale per Teare 2014 and Eldridge 2016. | p. 12–13 |
| 20. Recruitment | Via community partners and NHS staff networks. | p. 9–12 |
| 21a–21b. Sequence generation / type | Cluster randomisation (1:1) and stepped-wedge sequence pre-specified. | p. 9–13 |
| 22. Allocation concealment | Concealment not feasible; randomisation by independent researcher. | p. 10–12 |
| 23. Implementation | Staff assign clusters post-randomisation; consent obtained before allocation. | p. 10–12 |
| 24a–24c. Blinding | Unblinded design; objective feasibility endpoints reduce bias. | p. 10–12 |

| **Item** | **Description of How Addressed in Co-STARS Protocol** | **Page / Line Reference** |
| --- | --- | --- |
| 25a. Data collection methods | Validated scales (MAKS, RIBS, CAMI, GHSQ, CBMCS); observations + qualitative interviews. | p. 10–13 and 15–16 |
| 25b. Retention / follow-up | 3-week follow-up (WP1) and 4-week (WP2); contact through community / trust leads. | p. 10–12 and 15–16 |
| 26. Data management | Data management plan approved; secure servers, restricted access. | p. 16–17 |
| 27a–d. Statistical methods | Descriptive + ICC (Hemming 2017) using mixed-effects models; missing data explored, no imputation. | p. 12–15 |
| 28a. DMC / monitoring | No formal DMC; oversight by UoB Research Governance and Research Advisory Committee. | p. 16–17 |
| 28b. Interim analysis | No interim analyses; review after completion. | p. 12–15 |
| 29. Trial monitoring | Monitored by UoB Research Governance with periodic site meetings. | p. 16–17 |
| 30. Ethics approval | Approved by EoSRES (24/ES/0030, RG_23-166). | p. 16–17 |
| 31. Protocol amendments | Amendments submitted to sponsor and REC; registry updated. | p. 16–17 |
| 32a. Consent process | Written informed consent obtained by research staff or facilitators before data collection. | p. 16–17 |
| 32b. Additional consent | Participants directed to support services if distressed. | p. 16–17 |
| 33. Confidentiality | Personal data anonymised and stored securely under GDPR. | p. 16–17 |
| 34. Ancillary / post-trial care | Participants directed to NHS and community services if distressed. | p. 16–17 |
| 35. Trial status | Recruitment began March 2025; completion expected Oct 2025. | p. 7–9 and 16 |
