# Supplementary material for: Co-STARS: a feasibility evaluation of a co-produced mental health literacy training package to reduce mental health inequities for Black young people in underserved communities – study protocol for a randomised controlled trial with an external pilot, process evaluation and economic analysis
Source: BMJ Open. 2026 Mar 18;16(3):e103120. doi: 10.1136/bmjopen-2025-103120 (PMC13007151; doi:10.1136/bmjopen-2025-103120)
Supplement: Supplementary file 2 [file bmjopen-2025-103120.reviewer_comments.pdf]

PEER REVIEW HISTORY

BMJ Open publishes all reviews undertaken for accepted manuscripts. Reviewers are asked to complete a checklist review form and are provided with free text boxes to elaborate on their assessment. These free text comments are reproduced below.

ARTICLE DETAILS

Title (Provisional)

Co-Stars: A Feasibility Evaluation of a Co-Produced Mental Health Literacy Training Package to Reduce Mental Health Inequities for Black Young people in Underserved Communities: study protocol for a randomised controlled trial with an external pilot, process evaluation and economic analysis

Authors

Kumarendran, Balachandran; Cherrington, Elizabeth; Bandyopadhyay, Siddhartha; Crowe, Francesca; Machaczek, Katarzyna Karolina; Brown, Luke; Campbell, Niyah; Jordan, Gerald; Pope, Megan; Antonović, Milan; Taylor, Matthew; Nadia, Nadia; Mahamud, Ayan; Chandan, Joht Singh; Griffiths, Sian Lowri; Youth Co-production Group, Co-STARS

VERSION 1 - REVIEW

**Reviewer**	**1**
**Name**	**Fisher, Jane**
**Affiliation**	**Monash University, Jean Hailes Research Unit, School of Public Health and Preventative Medicine**
**Date**	**24-Jul-2025**
**COI**	**None**

This protocol is for a series of interlinked investigations using multiple methods to address inequities in experiences of psychosis among ‘Black’ young people in Birmingham through implementation of a training program. The goal is important, and the investigators have expertise in the serious mental health problems that are the focus of the proposal, and detailed knowledge of the setting and the local service structure. The co-design with young people with lived experience is a study strength.

The program of research is ambitious, but in my opinion, it would not be possible from this account to replicate the methods of the individual component studies, and it is difficult to understand how they are intended to inform each other or the overall goal. It is also quite parochial. This is a journal with a large international readership, but there are many

unexplained references to local social factors and services that will not be understandable to people outside the Birmingham area or England.

I think substantial revisions are needed, including:

1. If multiple studies are to be used, then it would be helpful to provide a conceptual framework explaining how they are related to each other. Although improving mental health literacy appears to be the mechanism of action, a theory of change to justify why each study is essential to the overall goal would assist. These should be added.

2. Is the aim prevention or early intervention or recovery, please explain.

3. This protocol refers to Phases, Tiers and Work Packages, but it is not clear how these relate to each other or whether they are different from each other. Perhaps the design could be simplified or explained in terms of all these elements.

4. The intention appears to be to improve the mental health literacy and cultural sensitivity of mental health professionals, but it is not clear how this might reduce the incidence of psychosis or assist with help seeking or recovery. This needs to be explained and the planned indicator of the outcomes provided.

5. Each study needs a crisp research question and a statement of the design that is appropriate to answer the question.

6. Each study needs a clear account of the inclusion and exclusion criteria for participants and how they will be recruited. These need to be added.

7. Each study needs a clear listing of the data sources, and their psychometric properties.

8. Each study needs an account of when the data will be collected and by whom, where the data will be stored and how they will be managed and analysed.

9. In WP1 what are the units of clustering and how will participants be recruited? How will the clusters be assigned to trial arms, and how will these be balanced as there is high potential with many types of cluster to introduce bias.

10. Was the content co-developed or only the study design?

11. How can cost-effectiveness be assessed outside a trial?

12. Readers cannot be presumed to know that the first phase of the authors' work involved, a clear explanation needs to be added.

13. As no effectiveness data are being sought, please explain why a trial is being conducted. The other outcomes could be ascertained in a simpler design.

14. Why are the outcomes not being assessed blind to group allocation? Please explain.

15. It is not clear what this sentence means: Parameters will be estimated for the power calculations for the secondary outcomes making adjustments for the temporal trend, ICC and number of steps. Please explain in plain language for general readers.

16. Please explain the following for readers who cannot be presumed to know the context in which this work is being conducted. Please explain all place-specific descriptions including:

• Not clear how 'Black', a term for skin colour, is an ethnicity. Please explain this

• Not clear how a prevalence rate can be described as a raw number (431.2). Also, can't have 0.2 of a person. Please explain what the denominator is and clarify these numbers.

• What is the 'top 10% of need' in England – please explain this descriptor for international readers.

• 'Building on our recent work' please describe this for readers who cannot be assumed to know that the recent work is.

• What is 'Forward Thinking Birmingham'?

• What are 'Black Country Early interventions'?

• What are 's136 interventions in Black males'?

**Reviewer**	**2**
**Name**	**Dack, Kyle**
**Affiliation**	**University of Bristol, MRC Integrative Epidemiology Unit**
**Date**	**18-Aug-2025**
**COI**	**None**

Statistical review summary:

This a protocol for an ambitious study project including multiple stages. While this protocol provides high-level details, it lacks specifics needed to ensure transparency and replicability – e.g. a full statistical analysis plan for each of the work packages. I have added some comments regarding specific details, but applying the SPIRIT checklist would be very useful as it is geared towards ensuring a clear, pre-specified statistical plan.

1. Reporting checklist: The protocol is generally comprehensive, but would still benefit from applying a reporting checklist to demonstrate this. The SPIRIT checklist (https://www.consort-spirit.org/) seems suitable as it is designed for protocols.

Abstract:

2. The abstract is clear and describes three stages of this study project. My primary suggestion is to consider adding more subheadings – currently there are only two, which makes it more difficult for abstract readers to navigate, e.g. finding the specifics of the intervention or outcomes.

3. The abstract mentions that a primary endpoint is “acceptability”, specifically for the first pilot trial. This is not a common outcome so I suggest explaining what it represents or how it will be quantified.

Introduction:

4. The key concepts, study population, and evidence gaps are all summarized clearly – no suggestions.

5. Aims and objectives – It is challenging to follow this because there are repeated subheadings (e.g. aims is a heading on pages 3 and 4), but my assumption is that everything from Line 35 Page 3 to Line 50 Page 4 is part of the aims/objectives section. I suggest the following to help clarify this section:

6. -Consider replacing “methods” on P3 with “project stage”, “study progress” or similar, because it is not really about methods.

7. -Are the “tier 1 / tier 2” labelling necessary? It seems to match to WP1 and WP2, and tier 1/2 naming is not used elsewhere in the paper.

8. -Consider moving the second “aims” section (P4 line 26) to before what is currently called “methods (P3 line 31). Because this “aims” section provides a high-level overview of the study phases and WP, giving a general introduction to the study plan. Whereas the information in the “methods” section describes the previous Phase 1 and the planned outputs which seem specifically relevant to WP1 and 2.

9. WP1: This is described as a cluster RCT – but what are the clusters? This should be described when clusters are first mentioned in the “methods” subheading.

10. WP1 units of randomization: How will clusters be determined – is there a pre-specified list?

11. WP1 outcome: “completion of pre- and post- outcome measures” – which outcomes are these, and how will they be measured? “acceptability” is listed as an outcome but it is unclear how this will be measured.

12. WP2: Randomization – this section currently describes the timing of crossovers but not the randomization : how is the randomization applied?

13. WP2 outcome: Similar to prior comments “acceptability” is listed (and referred to WP1) but there are not details of what this represents or how it is quantified.

14. WP2 analysis: This section is very brief and it is not clear how each of the three outcomes (training uptake, acceptability, and CBMCS survey) will be (a) quantified and (b) estimated. Which are the comparisons groups, software used, and how exactly will the outcomes be reported? SPIRIT may help identify more details, but generally I feel this section lacks replicability.

15. Estimating ICC: which software will be used for this, and which specific methods? How will the results be interpreted? There is mention of using this to identify outcomes most sensitive to change and inform sample size estimation but how specifically will this be applied?

16. Missing data is not mentioned – will this be checked, and what procedure will be applied if data is missing?

17. WP4: What are the specific outcomes which will be used to evaluate this part of the project? There are two analyses mentioned, but it is not clear how certainty will be estimated using these.

VERSION 1 - AUTHOR RESPONSE

Reviewer: 1 Prof. Jane Fisher, Monash University Comments to the Author:

This protocol is for a series of interlinked investigations using multiple methods to address inequities in experiences of psychosis among 'Black' young people in Birmingham through implementation of a training program. The goal is important, and the investigators have expertise in the serious mental health problems that are the focus of the proposal, and detailed knowledge of the setting and the local service structure. The co-design with young people with lived experience is a study strength.

The program of research is ambitious, but in my opinion, it would not be possible from this account to replicate the methods of the individual component studies, and it is difficult to understand how they are intended to inform each other or the overall goal. It is also quite parochial. This is a journal with a large international readership, but there are many unexplained references to local social factors and services that will not be understandable to people outside the Birmingham area or England.

I think substantial revisions are needed, including:

1. **Comment:** If multiple studies are to be used, then it would be helpful to provide a conceptual framework explaining how they are related to each other. Although improving mental health literacy appears to be the mechanism of action, a theory of change to justify why each study is essential to the overall goal would assist. These should be added.

Response: We thank the reviewer for this helpful suggestion. We have now added further detail around our conceptual framework and an accompanying theory of change to clarify how the work packages are interrelated and contribute to the overall goal of Co-STARS.

The overarching framework through which we examine *how, why, for whom, and under what circumstances* the Co-STARS interventions may generate system-wide change. Specifically, WP3 adopts a realist-informed mixed-methods approach—combining participatory systems mapping, Delphi consensus, and exploratory time-series analysis—to identify and test the mechanisms that connect improved mental health literacy and trust-building (the central mechanisms of change) to broader outcomes such as earlier help-seeking and reduced disparities in access and outcomes.

We have incorporated this conceptual framework and theory of change into the revised manuscript (see updated WP3 within the Methods and Overview of Work Packages sections). Together, these additions clarify the interrelationships between the WPs and demonstrate how each contributes to the overall aim of fostering earlier, fairer, and more effective access to mental health support for Black youth.

2. **Comment:** Is the aim prevention or early intervention or recovery, please explain.

Response: Thank you for this important question. The primary aim of the Co-STARS project is early intervention, with a strong preventive orientation. Specifically, Co-STARS focuses on promoting earlier identification and engagement with mental health support among Black youth by addressing key barriers such as stigma, mistrust, and inequitable access to care. By fostering community trust and mental health literacy, the project seeks to intervene before mental health challenges become more severe or entrenched, thereby preventing escalation and promoting long-term wellbeing. While recovery is an important downstream outcome, the central emphasis of Co-STARS is on earlier recognition and response to emerging mental health needs, positioning it primarily as an early intervention and prevention initiative.

3. **Comment:** This protocol refers to Phases, Tiers and Work Packages, but it is not clear how these relate to each other or whether they are different from each other. Perhaps the design could be simplified or explained in terms of all these elements.

Response: We thank the reviewer for this suggestion. In response, we have revised the Methods section to focus specifically on Phase 2 (the current study) to improve clarity and readability. We have also removed references to tiers to further simplify the presentation. Additionally, we have included an initial section outlining the overall phased nature of the program and evaluations, clarifying how the phases and work packages relate to each other. This restructuring simplifies the presentation of the study design while retaining the necessary context for understanding Phase 2.

4. **Comment:** The intention appears to be to improve the mental health literacy and cultural sensitivity of mental health professionals, but it is not clear how this might

reduce the incidence of psychosis or assist with help seeking or recovery. This needs to be explained and the planned indicator of the outcomes provided.

Response: We thank the reviewer for this valuable comment. We have clarified how the intervention mechanisms are expected to influence early help-seeking, access, and recovery through improved mental health literacy and culturally responsive care. A new explanatory paragraph has been added under the Introduction to link improved literacy and cultural competence with early intervention pathways and measurable outcomes.

5. **Comment:** Each study needs a crisp research question and a statement of the design that is appropriate to answer the question.

Response: We appreciate the reviewer's suggestion. Each work package now begins with a concise research question and study design statement to aid clarity and replicability.

6. **Comment:** Each study needs a clear account of the inclusion and exclusion criteria for participants and how they will be recruited. These need to be added.

Response: We agree and have expanded each work package's "Population" paragraph to provide explicit inclusion and exclusion criteria and recruitment processes.

7. **Comment:** Each study needs a clear listing of the data sources, and their psychometric properties.

Response: We thank the reviewer for this observation. We have now summarised data sources and referenced the reliability and validity of the psychometric instruments used in each work package.

8. **Comment:** Each study needs an account of when the data will be collected and by whom, where the data will be stored and how they will be managed and analysed.

Response: We thank the reviewer for highlighting the need for greater detail on data collection, management, and timing. We have clarified these elements under the Methods and Ethics and Dissemination sections.

9. **Comment:** In WP1 what are the units of clustering and how will participants be recruited? How will the clusters be assigned to trial arms, and how will these be balanced as there is high potential with many types of cluster to introduce bias.

Response: Middle Layer Super Output Areas (MSOAs) within Birmingham and the Black Country will serve as the units of randomisation (clusters) and will be randomly allocated on a 1:1 basis to receive the intervention or control. Each MSOA represents a geographically defined area with a population of approximately 5,000–15,000 residents, providing an appropriate ecological unit for community engagement and potential linkage with routinely collected data in future phases.

Within each selected MSOA, the intervention will be implemented in community settings such as places of worship (for example, Black-majority churches), youth centres, community centres, and youth residential settings that already serve local Black communities.

To ensure feasibility and representativeness, we restricted the sampling frame for cluster randomisation to MSOAs with intermediate Black population density, based on the proportion of residents identifying as Black (African, Caribbean, or Black British) in the 2021 Census. This restriction avoids areas where community engagement would be unfeasible (very low-density MSOAs) and reserves very high-density MSOAs for the future definitive trial. Importantly, if recruitment and delivery are feasible in intermediate-density MSOAs, it is likely to remain feasible in MSOAs with higher Black population density, providing a conservative test of feasibility.

Randomisation will use restricted (covariate-constrained) allocation to maintain approximate balance on (i) area-level deprivation and (ii) anticipated participant numbers (based on venue capacity and local population). The randomisation sequence will be generated by an independent researcher/statistician prior to implementation.

By design, concealing treatment allocation is not possible in this community-based feasibility study. Findings from this feasibility phase will inform sampling, stratification, and power calculations for the definitive trial.

We have updated the ‘Unit of Randomisation’ section in the Methods to include the details described above.

10. **Comment:** Was the content co-developed or only the study design?

Response: We thank the reviewer for this question. Both the content and study design were co-developed with young people from Black African and Black Caribbean backgrounds and community partners. We have clarified that co-development extended to the training materials, delivery format, and evaluation tools in addition to study design.

11. **Comment:** How can cost-effectiveness be assessed outside a trial?

Response: We thank the reviewer for this important methodological point. We have clarified that the current phase involves a feasibility-stage economic evaluation, which aims to test cost-data collection procedures and to produce preliminary cost-consequence estimates but not to evaluate effectiveness.

12. **Comment:** Readers cannot be presumed to know that the first phase of the authors' work involved, a clear explanation needs to be added.

    Response: We agree. A clarifying paragraph summarising Phase 1 outputs and their link to Phase 2 has been added to the Introduction before the Methods and Analysis section.

13. **Comment:** As no effectiveness data are being sought, please explain why a trial is being conducted. The other outcomes could be ascertained in a simpler design.

    Response: We thank the reviewer for this useful observation. We have clarified that this study adopts a feasibility trial design to test randomisation, recruitment, acceptability, and implementation processes required for a future definitive RCT-rather than to evaluate effectiveness at this stage.

14. **Comment:** Why are the outcomes not being assessed blind to group allocation? Please explain.

    Response: We acknowledge this important point. Because the interventions involve community-delivered training and e-learning within service teams, blinding of participants or researchers is not feasible. However, feasibility and process outcomes are objective and low-risk, reducing bias. We have added an explanatory note to both WP1 and WP2 Methods.

15. **Comment:** It is not clear what this sentence means: Parameters will be estimated for the power calculations for the secondary outcomes making adjustments for the temporal trend, ICC and number of steps. Please explain in plain language for general readers.

Response: We thank the reviewer for highlighting the need for clearer explanation. This sentence has been revised to provide a plain-language description of how estimates from this feasibility phase will inform the design of the definitive trial.

Specifically, we will calculate summary statistics (e.g., means, variances, and intra-cluster correlation coefficients [ICCs]) for the key outcome measures collected before and after the intervention across clusters and over time. These estimates will be used to understand how much outcomes vary within and between teams and how results might change across different time periods (temporal trends) and step transitions in the stepped-wedge design. These parameters are needed to perform accurate sample size and power calculations for a larger definitive trial.

Accordingly, the revised manuscript text now reads:

"We will estimate key parameters—such as variability of outcome measures, intra-cluster correlation coefficients (ICCs), and potential changes over time—to inform sample size and power calculations for a future definitive trial. These estimates will account for both the temporal trend and the number of step transitions in the stepped-wedge design."

16. **Comment:** Please explain the following for readers who cannot be presumed to know the context in which this work is being conducted. Please explain all place-specific descriptions including:

Not clear how 'Black', a term for skin colour, is an ethnicity. Please explain this.

Response: We thank the reviewer for this valid point. We agree that "Black" is not an ethnicity per se, and we do not intend to refer to it as such. Rather, we acknowledge the broadness and social complexity of this categorisation. Our intention was to capture the shared experiences of marginalisation and discrimination commonly faced by individuals of African and African-diaspora backgrounds, which are often grouped under the term "Black" in research and policy contexts.

Importantly, our young Black participants reflected on this throughout the co-production process. These reflections informed discussion points within the culturally responsive training, which emphasises that there is no single Black culture and that the intersections of an individual's social identities should be recognised and understood.

We now include the following section within the manuscript:

"Co-production and Cultural Considerations

Young people from Black African and Black Caribbean-diaspora backgrounds were actively involved throughout the co-production of this study. Discussions with participants highlighted that "Black" is not a single ethnicity or culture, and experiences vary widely depending on the intersections of social identities. These reflections

informed the development of culturally responsive training, emphasising the diversity within Black communities and the importance of recognising intersecting social identities. The co-production process ensured that the study design, measures, and materials were sensitive to participants' cultural and social contexts."

Comment: Not clear how a prevalence rate can be described as a raw number (431.2). Also, can't have 0.2 of a person. Please explain what the denominator is and clarify these numbers.

Response: We thank the reviewer for this observation. We have clarified that the figure (431.2) represents an estimated annual incidence rate per 100,000 people aged 16–35 years, based on modelled data from the PsyMaptic-A tool, rather than a count of individuals. We have rephrased the text to avoid implying fractional people.

Comment: What is the 'top 10% of need' in England – please explain this descriptor for international readers.

Response: We agree this term requires clarification. We have expanded it to explain that it refers to neighbourhoods falling within the most deprived decile of the English Index of Multiple Deprivation (IMD), which is widely used in UK public health research.

Comment: 'Building on our recent work' please describe this for readers who cannot be assumed to know that the recent work is.

Response: We thank the reviewer for this point. We have replaced the vague phrase "building on our recent work" with a clear description of the previous phase (Phase 1), summarising its purpose and outputs.

Comment: What is 'Forward Thinking Birmingham'? What are 'Black Country Early interventions'?

Response: We have now clarified that these are mental health trusts, and we now direct the reader to 'Setting' section, page 7, where a full description of these services are provided.

Comment: What are 's136 interventions in Black males'?

Response: We thank the reviewer for noting this ambiguity. We have clarified that "s136 interventions" refers to detentions under Section 136 of the UK Mental Health Act, which allows police officers to remove an individual appearing to have a mental disorder from a public place to a place of safety for assessment. We have revised the text to explain this term for international readers.

Reviewer: 2 Dr. Kyle Dack, University of Bristol Comments to the Author:

Statistical review summary: This a protocol for an ambitious study project including multiple stages. While this protocol provides high-level details, it lacks specifics needed to ensure transparency and replicability – e.g. a full statistical analysis plan for each of the work packages. I have added some comments regarding specific details, but applying the SPIRIT checklist would be very useful as it is geared towards ensuring a clear, pre-specified statistical plan.

1. **Comment:** Reporting checklist: The protocol is generally comprehensive, but would still benefit from applying a reporting checklist to demonstrate this. The **SPIRIT checklist** (https://www.consort-spirit.org/) seems suitable as it is designed for protocols.

Response: We thank the reviewer for this helpful recommendation. We have now completed and included the SPIRIT 2025 checklist with our revised submission to ensure full transparency and alignment with current reporting standards for trial protocols. Each item in the checklist cross-references the relevant page and line numbers in the manuscript. This addition demonstrates that all required methodological details are included and clearly located within the text.

Abstract:

2. **Comment:** The abstract is clear and describes three stages of this study project. My primary suggestion is to consider adding more subheadings – currently there are only two, which makes it more difficult for abstract readers to navigate, e.g. finding the specifics of the intervention or outcomes.

Response: Thank you for this feedback. We have formatted the abstract in line with the journal's formatting guidelines which requires the following subheadings: sections. Introduction; Methods and analysis; Ethics and dissemination.

3. **Comment:** The abstract mentions that a primary endpoint is "acceptability", specifically for the first pilot trial. This is not a common outcome so I suggest explaining what it represents or how it will be quantified.

Response: We thank the reviewer for highlighting this. We have now clarified in the abstract and methods that acceptability refers to participants' perceptions of the training's relevance, usefulness, and delivery, assessed through quantitative completion rates and qualitative feedback (focus groups and interviews). This aligns with the AIM (Acceptability of Intervention Measure) framework and process evaluation standards for feasibility trials.

Introduction:

The key concepts, study population, and evidence gaps are all summarized clearly – no suggestions.

5. **Comment:** Aims and objectives – It is challenging to follow this because there are repeated subheadings (e.g. aims is a heading on pages 3 and 4), but my assumption is that everything from Line 35 Page 3 to Line 50 Page 4 is part of the aims/objectives section. I suggest the following to help clarify this section:

   Consider replacing "methods" on P3 with "project stage", "study progress" or similar, because it is not really about methods.

Response: Thank you for this suggestion. We have amended this to 'methods and analysis' in-line with the journal's formatting guidelines and editor's suggestions. We also include a sentence on the status/progress of the study.

6. **Comment:** Are the "tier 1 / tier 2" labelling necessary? It seems to match to WP1 and WP2, and tier 1/2 naming is not used elsewhere in the paper.

Response: Thank you for your observation. To aid clarity and simplify the methods, we have removed the term 'tier' from the manuscript. -Consider moving the second "aims" section (P4 line 26) to before what is currently called "methods (P3 line 31). Because this "aims" section provides a high-level overview of the study phases and WP, giving a general introduction to the study plan. Whereas the information in the "methods" section describes the previous Phase 1 and the planned outputs which seem specifically relevant to WP1 and 2.

Thank you for this suggestion. We have created a separate section for overarching objectives and now only include methods relating specifically to our Phase 2 work within the 'Methods and analysis' section.

7. **Comment:** WP1: This is described as a cluster RCT – but what are the clusters? This should be described when clusters are first mentioned in the "methods" subheading.

Response: We thank the reviewer for highlighting this point. The manuscript has been revised to clarify that Middle Layer Super Output Areas (MSOAs) within Birmingham and the Black Country are the units of randomisation (clusters), while community settings within each MSOA (e.g., Black-majority churches, youth centres, and community centres) serve as the sites of intervention delivery. This clarification now appears when the trial design is first described under the Methods and Analysis section, ensuring consistency and transparency.

8. **Comment:** WP1 units of randomization: How will clusters be determined – is there a pre-specified list?

   Response: We appreciate the reviewer's request for clarification. Yes, there will be a pre-specified list of eligible clusters generated prior to randomisation.
   Clusters are defined as Middle Layer Super Output Areas (MSOAs) within Birmingham and the Black Country that fall within the restricted sampling frame (i.e., MSOAs with intermediate Black population density based on 2021 Census data). From this list, MSOAs will be randomly allocated (1:1) to the intervention or control arm using restricted (covariate-constrained) randomisation to maintain approximate balance on area-level deprivation and anticipated cluster size.

9. **Comment:** WP1 outcome: "completion of pre- and post- outcome measures" – which outcomes are these, and how will they be measured? "acceptability" is listed as an outcome but it is unclear how this will be measured.

   Response: We thank the reviewer for this helpful point. We have now specified which outcome measures will be administered pre- and post-intervention and clarified how acceptability will be assessed.

   Quantitative outcomes (knowledge, stigma, and help-seeking) will be assessed using validated scales (MAKS, RIBS, CAMI, and GHSQ). Acceptability will be evaluated both quantitatively—via participation, retention, and completion rates—and qualitatively through focus groups and interviews guided by the Acceptability of Intervention Measure (AIM) framework.

   These clarifications have been added to the Outcome paragraph of WP1.

10. **Comment:** WP2: Randomization – this section currently describes the timing of crossovers but not the randomization : how is the randomization applied?

    Response: We thank the reviewer for highlighting this point. We have now clarified how randomisation will be applied in the stepped-wedge design.
    All six participating clusters (clinical teams) will be identified in advance—four within Forward Thinking Birmingham and two within Black Country Healthcare NHS Foundation Trust.

    The order in which clusters cross over from the control to the intervention phase will be determined using a computer-generated random sequence, produced by a

member of the research team who is not involved in data collection, to ensure independence between allocation and assessment.

Given the small number of clusters, no stratification or balancing factors will be used. Each cluster will cross over once at approximately four-week intervals until all have received the intervention.

Comment: WP2 outcome: Similar to prior comments "acceptability" is listed (and referred to WP1) but there are not details of what this represents or how it is quantified. Please see response to point 3 above.WP2 analysis: This section is very brief and it is not clear how each of the three outcomes (training uptake, acceptability, and CBMCS survey) will be (a) quantified and (b) estimated. Which are the comparisons groups, software used, and how exactly will the outcomes be reported? SPIRIT may help identify more details, but generally I feel this section lacks replicability.

Response: We thank the reviewer for this constructive feedback. We have clarified the operationalisation and analysis of all WP2 outcomes.

Training uptake will be quantified as the proportion of eligible staff completing the e-learning module within each cluster.

Acceptability will be assessed quantitatively through training completion and satisfaction scores and qualitatively via focus groups and interviews, using the Acceptability of Intervention Measure (AIM) framework.

CBMCS outcomes will be analysed as mean pre–post changes in multicultural competence scores at individual and cluster levels.

For transparency, we have specified the comparison approach, analytic methods, and software: descriptive statistics and mixed-effects models using Stata 18. The revised text now provides sufficient detail for replication, in line with the SPIRIT 2025 and CONSORT-SW-CRT extensions.

11. **Comment:** Estimating ICC: which software will be used for this, and which specific methods? How will the results be interpreted? There is mention of using this to identify outcomes most sensitive to change and inform sample size estimation but how specifically will this be applied?
Response: We thank the reviewer for this important methodological point. We have clarified how the intra-cluster correlation coefficient (ICC) will be estimated and used.

Consistent with methodological recommendations by Prof. Karla Hemming (University of Birmingham) and colleagues, ICCs for each quantitative outcome will be estimated

using mixed-effects models with random intercepts for clusters. Analyses will be conducted using Stata (version 18), which provides unbiased ICC estimates under these models.

The ICC estimates, together with observed variances, completion rates, and temporal trends, will be used to:

- Identify which outcome measures demonstrate sufficient responsiveness (sensitivity to change) to be prioritised as primary endpoints in the definitive trial; and

- Generate realistic parameter estimates for sample size and power calculations, following Hemming et al.'s recommended approach for feasibility and pilot cluster trials.

12. **Comment:** Missing data is not mentioned – will this be checked, and what procedure will be applied if data is missing?

Response: We thank the reviewer for highlighting this important point. We have now clarified how missing data will be assessed and handled.

As this is a feasibility study, our primary aim is to assess the extent and patterns of missing data rather than to impute outcomes for hypothesis testing. We will report the proportion and distribution of missing values for each outcome and explore whether data are missing completely at random or systematically (e.g., by site or participant characteristics).

If levels of missingness are substantial, we will conduct sensitivity analyses using simple imputation methods (e.g., mean or regression-based imputation) to evaluate their potential impact on feasibility estimates.

This approach is consistent with good practice guidance for pilot and feasibility trials (Eldridge et al., Stat Methods Med Res, 2016; Teare et al., Trials, 2014).

13. **Comment: WP4**: What are the specific outcomes which will be used to evaluate this part of the project? There are two analyses mentioned, but it is not clear how certainty will be estimated using these.

Response: We thank the reviewer for this valuable comment.

We have now specified the primary and secondary economic outcomes and explained how uncertainty will be addressed.

The primary outcome will be incremental cost per improvement in mental health literacy and professional multicultural competence, as measured in WP1 and WP2. Secondary outcomes will include quality-of-life gains, service use reductions, and social return on investment (SROI).

Uncertainty in cost and outcome estimates will be assessed through deterministic and probabilistic sensitivity analyses and expressed as 95% confidence intervals and cost-effectiveness acceptability curves (CEACs). These additions enhance clarity and replicability.

VERSION 2 - REVIEW

**Reviewer**	**2**
**Name**	**Dack, Kyle**
**Affiliation**	**University of Bristol, MRC Integrative Epidemiology Unit**
**Date**	**10-Nov-2025**
**COI**	

This is a multi-project protocol and as such it is a challenge to include all details, but the current revision satisfies SPIRIT requirements and the changes have addressed all my prior queries.
